# Clinical Lecture

**Published:** 1870-08

**Authors:** Jno. E. Owens

**Affiliations:** Surgeon to the Hospital; 112 Randolph Street


ARTICLE V.—*Clinical Lecture.* Delivered at St. Luke's Hospital. By JNO. E. OWENS, M.D., Surgeon to the Hospital. (*Thrombosis and Aphasia.*)

(Continued from last Number.)

Now, gentlemen, having finished our remarks upon the leg, let us turn our attention to other symptoms, not less interesting, namely, the paralysis, which manifested itself on the right side, the sixth day after the occurrence of the pain in the foot and leg, and the patient's inability to give expression to his thoughts. The first is called hemiplegia, which, as a symptom, is familiar to you, and of which I need say very little. The second, (impaired articulation) has been designated by several names, but I will only burden your minds with that which seems the best—Aphasia. The word itself means want of speech, and the condition of an aphasic, is, for the most part, one of inability to express thoughts by words, by writing and by gestures. There seems also to be some impairment of the intelligence. There is generally a transitory hemiplegia, which, where it does exist, is almost always on the right side. The symptoms—the paralysis, and the defect in articulation—come on suddenly. Let us see how far the phenomena, in our case, will agree with this definition. You will remember, that he called the nurse at six o'clock, A. M., asking him if he did not intend to get up soon. A few minutes afterwards, the nurse, upon going to the ward, discovered that the patient mumbled his words so as not to be understood. At the morning visit, (half-past ten o'clock,) the right arm and leg, and the right side of the face, were almost completely paralyzed. The muscles of the left side of the face, being intact, drew the corresponding corner of the mouth towards that side. The patient was unable to thrust the tongue from the mouth. The paralysis of the leg was well nigh complete. He was perfectly conscious. This condition of paralysis, you observed, prevented motion on the right side, and, at this stage, the paralysis of the tongue accounted for the inability to speak.

The paralysis, occurring Sunday, October 7th, had disappeared by Wednesday, October 10th, leaving the arm and leg weakened for a day or two longer. Although at this last date, he could thrust out the tongue and move it in every direction, his articulation was sadly impaired, and he had only several words at his command. "That'll do," "No," and "Yes," composed his vocabulary. You will remember that he used "No" occasionally, and only twice, I believe, did he employ "That'll do." "Yes," seemed to be the favorite, and it was generally repeated rapidly three times in succession—"yes, yes, yes." The word was more frequently an incorrect than a correct answer to the question asked. For instance to the following questions:—"How are you?" "have you much pain?" "what day is this?" "when were you admitted to the hospital?" the common answer was "yes, yes, yes." It seems, then, that when "yes" was a correct answer, it was also incidental, and expressed nothing intelligent. You will remember also, the patient's inability to tell his age. He never could articulate more than "thir," which is but a part of a word, yet he manifested, in his expression, very evident vexation, because of his inability to answer perfectly. When told that he was forty years old, he answered, "no." When told that he was thirty-two, with an expression of satisfaction, he answered, "yes." Hence we must conclude, that this word, which generally signified nothing, could be made an expression of intelligence.

You will also remember that the patient was unable to tell us his birth-place (England), after he became aphasic. And although when told that he was born in France or Germany, he replied, "no," he answered "yes" when England was mentioned. This shows that he knew, perfectly well, his birth-place, and yet except in this peculiar manner, could give us no information upon that point. He could not articulate the word England, any more than he could say France or Germany, or tell us his name. The patient was not even so successful in writing as in speaking. For he was unable to write a single legible word with either hand. Wherever I have known the experiment tried, in health, one, who can write with the right hand, can, with care, write a single word, at least, with the left hand. His attempts to write, resembled, very much, the tracings of that grand humbug—"La Planchette." He always, after attempting to write, threw the slate aside, manifesting the most marked expression of vexation. I suppose we may be allowed to include expression amongst gestures. If so, you will perceive that the patient was as limited in gesture, as he was in speech. Although, on several occasions, the patient manifested vexation in his expression, yet when asked to simulate an expression of vexation he was unable to do so.

You must not suppose that all cases of Aphasia are exactly like this one. The symptoms are varied and curious. Certain patients, whose modes of expression are as limited as those of the case under consideration, appreciate the relative value of cards, play a good game, and exult over success. An aphasic, a Frenchman, who could articulate "bonjour" could not be made to say bonbon—a mere repetition of the first syllable.

Again, Aphasia frequently sets in without paralysis; there is another class, in which the hemiplegia is but slightly marked and transitory; and a third class, in which the paralysis is more persistent. The prognosis is the most unfavorable in the latter class, because, in this, the brain lesion is the most extensive—the most deeply seated. There is another curious point in connection with this affliction, namely, the paralysis, when it does exist, is almost invariably on the right side. Now what must have been the cause of Aphasia which was persistent, and the well marked hemiplegia which came suddenly, and which disappeared in three days? Hemorrhage into the brain substance does not produce paralysis, so nearly complete as this, without coma. In sudden and complete or nearly complete hemiplegia, with coma, you may be pretty sure of cerebral hemorrhage. Sudden paralysis, complete or nearly complete, without coma, is ordinarily due to the softening of the brain. I felt sure, in our case, that a thrombus had occurred in one of the cerebral arteries going to the left side of the brain; that the paralysis commencing at the time of the occurrence of the thrombosis, disappeared as the collateral circulation became established; that the Aphasia, which had a cause, in common with the paralysis, was kept up from the supervention of softening. Thrombi usually take place in the middle cerebral artery, as it passes through the fissure of Sylvius. An examination, however, of the arteries failed to confirm the diagnosis of thrombosis. It is possible that a thrombus may have occurred in some part of the internal carotid, as it passed through the base of the skull. The body was taken away for interment, soon after the brain was removed, and it was impossible to carry the examination any further. This seemed to me the best explanation of a hemiplegia, occurring suddenly, so nearly complete, but disappearing in three days. I should not have expected such a disappearance in case of softening alone. Yet you must not lose sight of the fact that this transitory hemiplegia belongs to a majority of the cases of Aphasia —cases in which neither thrombi or emboli exist. The theories referring to the causation of Aphasia, are, unfortunately, somewhat numerous. Not one of them, however, will withstand pathological research. Indeed, the pathology of this affection seems, as yet, undetermined. If we have one set of pathologists placing the organ for the manifestation of thought by speech in the two anterior lobes of the brain, an authentic case is recorded, where, in a duel, an officer was struck in the temple by a ball, which passed through the two frontal lobes, in their middle portion, and from the first day there had been neither paralysis or any hesitation in speech.* If we have a second set, locating the organ of themanifestation of thought by speech exclusively in the left hemisphere, an equally authentic case is recorded of Aphasia with left hemiplegia.* If we have a third set, locatingt his organ in the posterior part of the third left frontal convolution, a case is recorded, where the autopsy is made by an eminent observer, in conjunction with the originator of the latter theory, and where both macroscopical and microscopical examination forced the conclusion that the posterior part of the third frontal convolution w[illegible]s not diseased.* A fourth class have located this organ at the junction of the frontal and middle lobes of the left side. The case which disproves the second theory mentioned, also disposes of this one. We see, then, how very little, that is definite and reliable, has, as yet, been discovered concerning the pathology of Aphasia. We have gained very little information, on this point, from an examination of the brain, which, although removed in due time, was not examined for forty hours subsequently, at which time the whole brain was much softened. It was plainly to be seen, however, that the posterior part of the left frontal lobe, the insula of Reil, a small portion of the middle lobe, the corpus striatum, and probably the optic thalamus, were softer than the surrounding brain substance of the left side, and the corresponding parts of the right side. The autopsy, under

* Trosseau.

the circumstances, I think is not of very much value. Yet the whole case is very interesting. The field is open for the microscopist and physiologist, to whom we look for a better understanding of Aphasia.

112 Randolph Street.

